# Rehabilitation training combined acupuncture for limb hemiplegia caused by cerebral infarction: A protocol for a systematic review of randomized controlled trial

**DOI:** 10.1097/MD.0000000000023474

**Published:** 2021-01-08

**Authors:** Jinqiu Wang, Chunlong Ran, Ping Pan, Yingrui Wang, Yinglin Zhao

**Affiliations:** a Henan University of Chinese Medicine; b The Second Affiliated Hospital of Henan University of Traditional Chinese Medicine, Zhengzhou, Henan Province, P.R. China.

**Keywords:** acupuncture, cerebral infarction, effectiveness, limb hemiplegia, rehabilitation training, safety, systematic review

## Abstract

**Background:**

Previous studies have reported that rehabilitation training combined acupuncture (RTA) can be used for the treatment of limb hemiplegia (LH) caused by cerebral infarction (CI). However, its effectiveness is still unclear. In this systematic review study, we aim to evaluate the effectiveness and safety of RTA for LH following CI.

**Methods:**

We will retrieve the databases of CENTRAL, EMBASE, MEDILINE, CINAHL, AMED, CBM, PUBMED, and CNKI from inception to June 1, 2020 with no language restrictions. The randomized controlled trials of RTA for evaluating effectiveness and safety in patients with LH following CI will be included. Cochrane risk of bias tool will be used to measure the methodological quality for all included studies. Two authors will independently select the studies, extract the data, and assess the methodological quality of included studies. A third author will be invited to discuss if any disagreements exist between 2 authors. We will perform heterogeneity assessment before carrying out meta-analysis. According to the heterogeneity, we select random effect model or fixed effect model for meta-analysis of the included cohort studies. Cochrane risk of bias tool will be used to determine the methodological quality for included studies. RevMan 5.3 software (Cochrane Community, London, UK) will be utilized to perform statistical analysis.

**Results:**

This systematic review will assess the effectiveness and safety of RTA for LH caused by CI. The primary outcome includes limbs function, as measured by the Wolf Motor Function Test (WMFT) Assessment scale, or other associated scales. The secondary outcomes consist of muscle strength, muscle tone, quality of life, and any adverse events.

**Conclusion:**

The findings of this study will summarize the current evidence of RTA for LH caused by CI, and may provide helpful evidence for the clinical treatment.

**Dissemination and ethics:**

The findings of this study are expected to be published in peer-reviewed journals. It does not require ethical approval, because no individual data will be utilized in this study.

**Systematic review registration:**

INPLASY202070114.

## 1 Introduction

Cerebral infarction is the main pathogenesis of stroke, 8 of 10 strokes are due to cerebral infarction, two from cerebral hemorrhage. Stroke is the most common cause of disability, the second commonest cause of dementia and the fourth commonest cause of death in the developed world. The incidence of stroke is 150–200/100.000 individuals/ year. One of every seven individuals suffers from stroke in their lifetime.^[1]^ cerebral infarction, an ischemic stroke, more severely affects patients’ nutritional status than other diseases.^[2]^ Limb hemiplegia is one of the main clinical symptoms of cerebral infarction,^[3]^ and the conventional treatment effect is not significant. As much as 80% stroke patients have extremities hypokinesia in the acute phase and only 5% to 20% patients can recover extremities function even if accepting rehabilitation therapy.^[4,5]^

Although current clinically emergency interventions of lowering blood pressure and surgical craniotomy have been applied very well, many patients still experience LH after the treatment.^[6–8]^ Lots of physical therapies are reported to help LH recovery^[9–16]^ especially for acupuncture and rehabilitation training.^[9,13,17–26]^ However, no study has systematically evaluated the effectiveness and safety of rehabilitation training with acupuncture (RTA) for the treatment of LH after CI with higher level evidence. Therefore, this systematic review aims to assess the effectiveness and safety of RTA for the treatment of patients with LH following CI.

## 2 Objective

This systematic review aims to summarize the current evidence of RTA for LH caused by CI, and may provide helpful evidence for the clinical treatment.

## 3 Methods

### 3.1 Study registration

The protocol was registered on the International Platform of Registered Systematic Review and Meta-analysis Protocols (INPLASY202070114) which could be available on https://inplasy.com. The content refers to the statement of the Preferred Reporting Items for Systematic Review and Meta-Analysis Protocols (PRISMA-P) checklist.^[27]^

### 3.2 Ethics and dissemination

The protocol of this systematic review will be disseminated in a peer reviewed journal and presented at relevant conferences. It is not necessary for a formal ethical approval, because the data are not individualized.

### 3.3 Inclusion criteria for study selection

#### 3.3.1 Types of participants

Patients with LH following CI, regarding sex, age, and race will all be included. However, patients are diagnosed with LH before the CI, or result from other disorders, except the CI will be excluded.

#### 3.3.2 Types of Studies

Original studies of randomized controlled trials (RCTs) of RTA for the treatment of LH following CI will be included without publication status restriction or writing language letters to editors, review articles, case reports, conference abstracts, cross-sectional studies, and all observational studies will be excluded.

#### 3.3.3 Types of interventions and controls

Experimental interventions: The patients in the treatment group received rehabilitation training and acupuncture (no restriction on the methods of operation and course of treatment) and guideline-recommended conventional treatment.

Control: The control group could gain guideline-recommended conventional treatment and a placebo or no treatment.

### 3.4 Types of outcome measures

#### 3.4.1 Primary outcome

The primary outcome includes limbs function, as measured by the WMFT Assessment scale, or other associated scales.

#### 3.4.2 Secondary outcomes

The secondary outcomes include muscle strength, as assessed by the motricity index or other related score tools; muscle tone, as evaluated by modified Ashworth scale, or other relevant scales; and quality of life, as examined by activities of daily living scale or any other specific scales. In addition, adverse events are also assessed.

### 3.5 Search strategy

#### 3.5.1 Data sources

Electronic databases including English databases (PubMed, MEDLINE, EMBASE, Web of Science, Cochrane Library) and Chinese databases (China National Knowledge Infrastructure, China Biology Medicine Database, Wanfang Database, VIP Database) will be searched from their inception to July 2020 with no language restrictions to recognize related studies. The RCTs that evaluate the effectiveness and safety of RTA for LH caused by CI will be included. The search strategy that will be run in the PubMed and tailored to the other database when necessary is presented in Table 1. Besides, the reference lists of review articles will be searched for any possible titles matching the inclusion criteria. Similar strategies will be applied to the other electronic databases in this study.

**Table 1:** The initial draft of the search strategy with PubMed as an example.

Number	Search terms
1	Mesh descriptor: (cerebral infarction) explode all trees
2	Mesh descriptor: (cerebrovascular accident) explode all trees
3	Mesh descriptor: (infarction stroke) explode all trees
4	((cerebral infarction^∗^) or (cerebral^∗^) or (cerebral next infarction^∗^) or (infarction^∗^) or (infarction stroke^∗^) or (infarction apoplexy^∗^)):ti, ab, kw
5	Mesh descriptor: (extremities) explode all trees
6	Mesh descriptor: (hemiplegia) explode all trees
7	Mesh descriptor: (paralysis) explode all trees
8	((extremities^∗^) or (hemiplegia^∗^) or (paralysis^∗^) or (limb^∗^) or (limbs^∗^) or (extremities hemiplegia^∗^) or (extremities paralysis^∗^) or (limb paralysis^∗^) or (limb hemiplegia^∗^) or (limbs paralysis^∗^) or (limbs hemiplegia^∗^)):ti, ab, kw
9	Or 1-8
10	MeSH descriptor: (acupuncture) explode all trees
11	MeSH descriptor: (acupuncture therapy) explode all trees
12	((acupuncture^∗^) or (acupuncture therapy^∗^) or (therapy^∗^) or (manual acupuncture^∗^) or (electroacupuncture^∗^) o r (fire needling^∗^) or (warm needling^∗^) or (scalp acupuncture^∗^) or (auricular acupuncture^∗^) or (intradermal needling^∗^)):ti, ab, kw
13	Or 10-12
14	MeSH descriptor: (rehabilitation) explode all trees
15	MeSH descriptor: (education) explode all trees
16	((rehabilitation^∗^) or (education^∗^) or (training^∗^) or (rehabilitation training^∗^) or (rehabilitation education^∗^)):ti, ab, kw
17	Or 14-16
18	MeSH descriptor: (randomized controlled trials) explode all trees
19	MeSH descriptor: (controlled) explode all trees
20	MeSH descriptor: (clinical trialsl) explode all trees
21	((random^∗^) or (allocation^∗^) or (random allocation^∗^) or (placebo^∗^) or (single blind^∗^) or (double blind^∗^) or (randomized control trial^∗^) or (RCT^∗^) o r (clinical trials^∗^) or (controlled clinical trials^∗^)):ti, ab, kw
22	Or 18–21
23	9 and 13 and 17 and 22

#### 3.5.2 Searching other resources

The researchers will also scan the database of Henan University of Traditional Chinese Medicine Library and consult the experts in Neurology. Dissertations of degrees will be included. The WHO International Clinical Trials Registry Platform and Google Scholar will be scrutinized for potential results. In addition, the Clinical Trials govregistry will be explored for any unpublished trials.

### 3.6 Data collection and analysis

#### 3.6.1 Selection of studies

**Figure 1:**
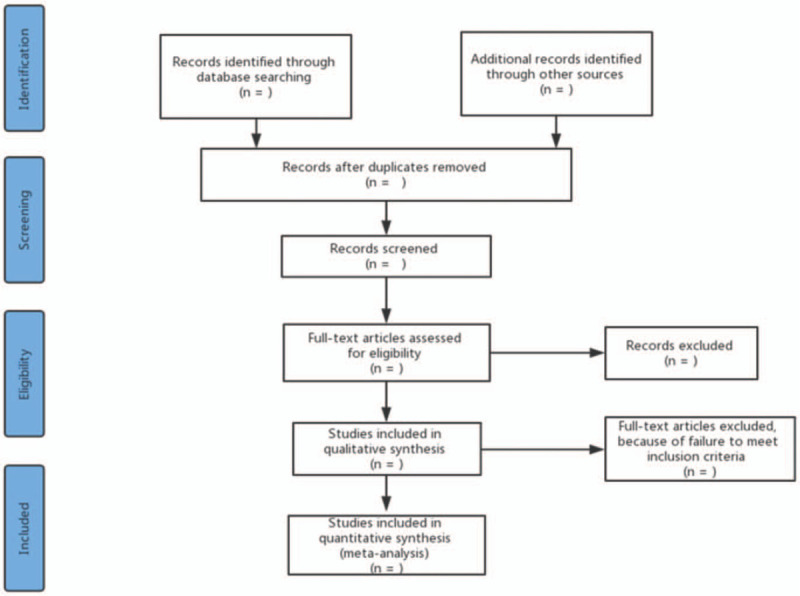
Flow diagram of study selection process.

Before the search begins, each reviewer will receive professional training to ensure consistency in the selection process and avoid the risk of bias (ROB) in human factors. According to pre-defined eligibility criteria, researchers will import the literature retrieved to the EndnoteX8 and eliminate the duplicate data. Studies will be removed if they do not meet the inclusion criteria obviously. If the studies appear to meet the inclusion criteria or there is any uncertainty based on the information provided in the title and abstract, full texts will be obtained for further assessment. When necessary, we will contact the author for more details of the study to solve questions about eligibility. Two researchers will independently conduct the literature search and literature screening. Disagreements will be resolved by discussion or taking the expert (DGC) for arbitration. The number and reasons for excluding trials will be recorded in detail. The details of selection process will be shown in the Preferred Reporting Items for Systematic Reviews and Meta-Analyses (PRISMA) flow chart Figure 1.

#### 3.6.2 Data extraction and management

Two researchers will extract data independently by using a predefined standardized form. The exacted information consists of general information (authors, year of publication, country, age, sex, ethnicity, disease types, diagnostic criteria, inclusion, and exclusion criteria); study design (sample size, details of randomization, allocation, and blinding); details of interventions (dosage, frequency, treatment duration); and outcome measurements (primary and secondary outcomes, adverse events, and any others). Any divergences between 2 researchers will be solved by a third researcher involving through consultation.

#### 3.6.3 Risk of bias evaluation

The risk of bias will be independently assessed by two reviewers and any differences will be resolved through consultation or the participation of a third reviewer. The RCTs will be evaluated using the Cochrane “risk of bias assessment” tool. The tool assesses the risk of bias mainly in the following 7 aspects: random sequence generation, allocation concealment, the blinding method for patients, researchers and outcomes assessors, incomplete result data, and selective reports. As recommended by the Cochrane manual, the risk of bias in each of these areas will be assessed as low or high depending on whether the criteria were met or were not met, and the lack of information will be recorded as unclear. In most cases, disagreements will be settled by discussion between the 2 reviewers. If disagreement remained after discussion, a third reviewer will be consulted before taking the final decision on the disagreements.

#### 3.6.4 Measurement of treatment effect

In this systematic review, continuous data will be presented as mean difference or standardized mean difference with 95% confidence intervals (CIs). The dichotomous data will be presented as the risk ratio with 95% CIs.

#### 3.6.5 Missing data management

If included study has missing, or insufficient or unclear data, those data will be required from the original authors by contracting them with emails. If those data will not get table, this study will only analyze the present available data, and it will be discussed in the text.

### 3.7 Quantitative data synthesis and statistical methods

#### 3.7.1 Data synthesis

We will use RevMan 5.3 software to carry out the data synthesis and meta-analysis. If heterogeneity is acceptable (*I*^2^ ≤ 50%), a fixed-effect model will be utilized to synthesize the data, and meta-analysis will be performed. In contrast, if heterogeneity is significant (*I*^2^ > 50%), a random-effect model will be used to pool the data and to operate the meta-analysis. In such a situation, subgroup analysis will be conducted to identify the factors that may cause the significant heterogeneity. If there is still substantial heterogeneity after the subgroup analysis, then data will not be pooled, and meta-analysis will not be conducted. Instead, a narrative summary will be described.

#### 3.7.2 Heterogeneity assessment

Tests of *I*^2^ and *x*^2^ will be applied to detect the possible heterogeneity. The heterogeneity will be considered as acceptable if the value of *I*^2^ is less than 50% (*I*^2^ ≤ 50%). Otherwise, significant heterogeneity will be considered if the value of *I*^2^ is more than 50% (*I*^2^ > 50%).

#### 3.7.3 Sensitivity analysis

A sensitivity analysis will be performed to test the robustness of the review result and to detect the source of heterogeneity. This can be done by excluding trials with a high risk of bias or eliminating each study individually. And, the impact of methodological quality, sample size, and missing data will be assessed. Then, the analysis will be repeated after the exclusion of low methodological quality studies and the results compared with the previous meta-analysis.

#### 3.7.4 Subgroup analysis

If significant heterogeneity will be detected, subgroup analysis will be carried out in accordance with the different treatments, control interventions, and outcome measurements.

#### 3.7.5 Grading the quality of evidence

Grading of Recommendations Assessment, Development and Evaluation (GRADE) method^[28]^ will be performed to evaluate the level of confidence in regards to outcomes. It is based on five key domains: risk of bias, consistency, directness, precision, and publication bias. Two independent reviewers will assess these studies. In most cases, disagreements were resolved by discussion between the 2 reviewers. If disagreement remained after discussion, the third reviewer will be consulted before taking the final decision on the disagreements.

#### 3.7.6 Meta-analysis

Meta-analysis will be performed by using RevMan (Review Manager, version 5.3). The HRs of the included studies will be combined by using both fixed-effects model and random-effects model, and we will check the consistency of the results between the 2 models. When *I*^2^ < 50%, we will report the result generated by the fixed-effects model; otherwise, we will choose the result by the random-effects model.

#### 3.7.7 Publication bias

Egger test will be used to assess the publication bias and the small study bias. When a *P*-value < 10 in the regression asymmetry is found, we will consider the existence of small-study effect. However, if there are <10 articles included, publication bias will not be explored.

#### 3.7.8 Trial sequential analysis

Meta-analysis may generate false-positive findings (type I error) because of repeated significance testing. To assess the risk of type I error, we will apply trial sequential analysis (TSA), which estimates the information size (cumulated sample size of the included studies) and adjusts threshold for statistical significance (TSA software, version 0.9 beta).

## 4 Discussion

The protocol of this systematic review will be conducted to assess the effectiveness and safety of RTA for the treatment of patients with LH following CI. As far as we know, no systematic review and meta-analysis have been addressed to investigate this issue. Therefore, it is very important to conduct this systematic review for assessing the effectiveness and safety of RTA for LH caused by CI.

In the present systematic review, we will search all related studies without language restrictions. All potential studies regarding the effectiveness and safety of RTA for LH following CI will be fully considered. The results of this study may present solid data to future research protocols, and also provide an up-to-date summary of the present evidence on the effectiveness and safety of RTA for patients with LH following CI. The findings of this study may also bring helpful evidence for clinicians.

## Author contributions

YLZ is the guarantor of the article. All review authors critically reviewed, revised, and approved the subsequent and final version of the protocol.

**Conceptualization:** Chunlong Ran, Yinglin Zhao.

**Data curation:** Jinqiu Wang, Chunlong Ran, Yinglin Zhao.

**Formal analysis:** Chunlong Ran.

**Methodology:** Chunlong Ran, Ping Pan, Yingrui Wang, Yinglin Zhao.

**Project administration:** Jinqiu Wang.

**Software:** Ping Pan.

**Supervision:** Yingrui Wang, Yinglin Zhao.

**Writing – original draft:** Jinqiu Wang.

**Writing – review & editing:** Jinqiu Wang, Chunlong Ran.
